# Increasing Evidence for the Association of Breast Implant-Associated Anaplastic Large Cell Lymphoma and Li Fraumeni Syndrome

**DOI:** 10.1155/2019/5647940

**Published:** 2019-07-16

**Authors:** Julian Adlard, Cathy Burton, Philip Turton

**Affiliations:** ^1^Yorkshire Regional Genetics Service, Chapel Allerton Hospital, Leeds, LS7 4SA, UK; ^2^Department of Haematology, St James's University Hospital, Leeds, LS9 7TF, UK; ^3^Department of Breast and Endocrine Surgery, St James's University Hospital, Leeds, LS9 7TF, UK

## Abstract

We report a case of breast implant-associated anaplastic large cell lymphoma (BIA-ALCL) occurring in a 53-year-old female with Li Fraumeni syndrome (LFS) with a prior history of breast cancer. We present the clinical features, investigation, and management of this patient and potential mechanisms that could explain the increasing association of BIA-ALCL and LFS.

## 1. Introduction

Breast implant-associated anaplastic large cell lymphoma (BIA-ALCL) is a rare T-cell lymphoma particularly associated with textured breast implants [1]. In 2016, the World Health Organization classification of lymphoid neoplasms included BIA-ALCL as a separate entity from other ALCLs, with features of CD30-positivity and* ALK*-negativity [2]. Assessing the incidence in women with textured implants has proved challenging, with estimates varying from 1 in 300,000 to 1 in 1000-10,000, with more recent reports suggesting the higher range [3]. National Comprehensive Cancer Network (NCCN) consensus guidelines have been published on diagnosis and treatment [4]. BIA-ALCL is generally associated with a good prognosis. However, concern regarding the association has led to the French National Agency for Safety of Medicines and Health Products (ANSM) recently withdrawing certain textured implant products from the market [5].

Li Fraumeni syndrome is an autosomal dominantly inherited multiple cancer predisposition syndrome due to germline pathogenic mutations in the* TP53* gene [6]. Typical malignancies associated with the syndrome include breast cancer, sarcomas, adrenocortical carcinoma, and primary brain tumours. The population prevalence has been estimated historically at around 1 in 20,000, although underascertainment and existence of lower-penetrance variants mean that the true penetrance is likely to be higher. A study including genomic datasets estimated a conservative prevalence of 1 in 3555-5476 and potentially as high as 1 in 400-865, if variants with less clear evidence for pathogenicity were included [7]. Screening and management protocols have been proposed for pathogenic mutation carriers, including whole body magnetic resonance imaging [8]. Imaging involving ionizing radiation and treatment with radiotherapy is advised to be minimised, or avoided unless essential, in view of the potential for inducing subsequent malignancies. Women with Li Fraumeni syndrome may consider risk-reducing bilateral mastectomies.

We report a case of BIA-ACLC occurring in a patient subsequently confirmed to have Li Fraumeni syndrome.

## 2. Case Report

The patient is a 53-year-old female. At the age of 33, she was diagnosed with grade 3, ER and PR negative, axillary node negative, ductal adenocarcinoma of the right breast cancer in the 2 o'clock position. HER2 status was not available, as testing was not routinely available at that time. This cancer was treated with wide local excision, axillary node clearance, adjuvant cyclophosphamide, methotrexate and fluorouracil chemotherapy, and radiotherapy to the right breast (40 Gy in 15 fractions, plus a boost to the tumour bed of 14 Gy in 7 fractions). Routine follow-up was with annual bilateral mammography. Three years subsequently, she developed two new tumours in the same breast in the 12 o'clock position; both were grade 3 ductal carcinomas ER and PR negative, and HER2 positive. She underwent right mastectomy and received adjuvant fluorouracil, epirubicin, and cyclophosphamide chemotherapy; trastuzumab treatment was not available at that time. In view of additional family history of breast cancer, BRCA1 and BRCA2 testing was performed, with no mutation detected. The patient elected to have a contralateral left risk-reducing mastectomy at the age of 36; histological examination of the left breast showed involutional fibrosis, fibroadenomatoid nodules, and duct dilatation, but no in situ or invasive malignancy. At the age of 38, she had bilateral breast reconstructions with subpectoral McGhan Style 150 Biocell textured expandable implants with remote ports, size range 385-405cc, with a gel volume of 125cc and saline fill range of 260-280cc (catalogue number 27-150836). Two years later, the right implant was replaced because of deflation with another of the same type.

**Figure 1:**
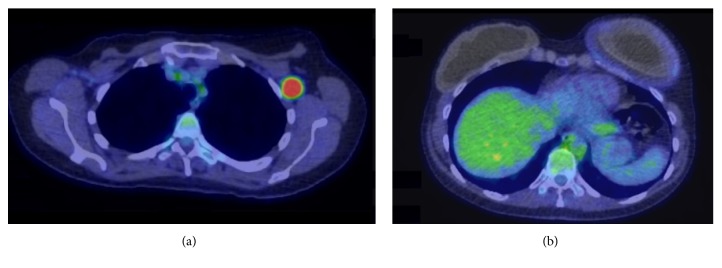
PET-CT scan images. (a) Pathologically enlarged, metabolically active left axillary lymph node. (b) Metabolically active thickening and seroma around left breast implant.

**Figure 2:**
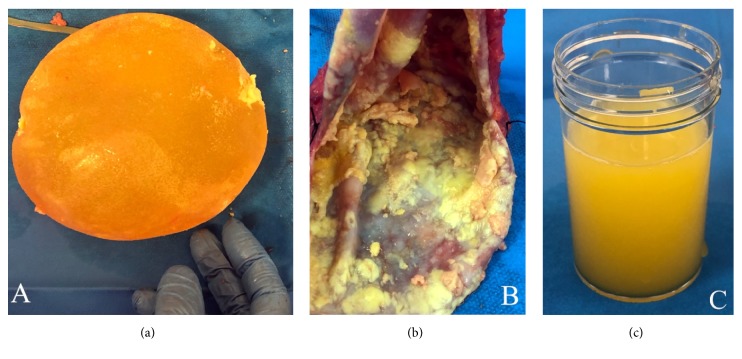
(a) Surgically removed textured silicone implant. (b) Fibrous capsule from which the implant was removed; solid BIA-ALCL deposits on the internal surface; (c) seroma fluid from inside the fibrous capsule which contained CD30-positive atypical lymphocytes.

**Figure 3:**
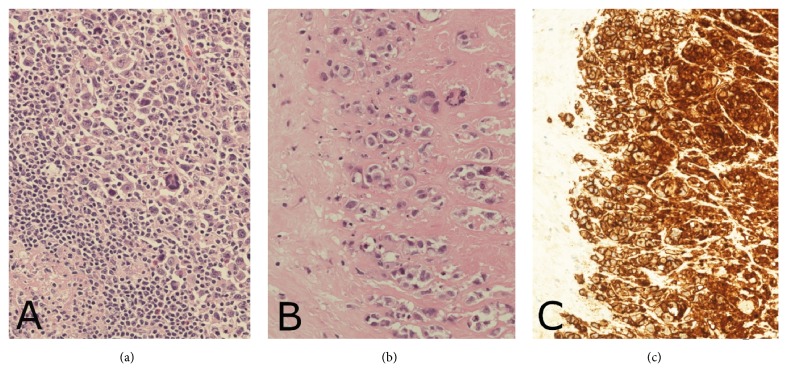
(a) H&E axillary lymph node showing ALCL; (b) H&E capsule showing BIA-ALCL; (c) CD30 positive immunohistochemical staining on capsular lymphoma cells.

At the age of 52, she presented with concerns regarding gradual-onset firm swelling of the left reconstructed breast over 12 months, with subsequent development of a separate easily palpable lump in the left axilla. Ultrasound and CT scans demonstrated a pathologically enlarged left axillary lymph node, approximately 20 mm diameter, and fluid around the left implant up to 19 mm in depth. PET CT scan confirmed increased uptake in the left axillary node and around the left breast implant (Figure 1), without other evident areas of disease. Both implants appeared intact on MRI scan. Fine needle aspiration cytologies from the left axillary node and from the left peri-implant fluid were both consistent with high-grade lymphoma. Surgery was performed with en bloc capsulectomy and removal of the implant (Figure 2). The left axilla was explored, where multiple abnormal lymph nodes were identified at anatomical level one. The largest lymph node was excised. Histopathological examination (Figure 3) demonstrated both the breast capsule and lymph node to be infiltrated with CD30 positive,* ALK*-negative BIA-ALCL. This was somewhat unusual in showing lymph node involvement and also expression of CD15.

**Figure 4:**
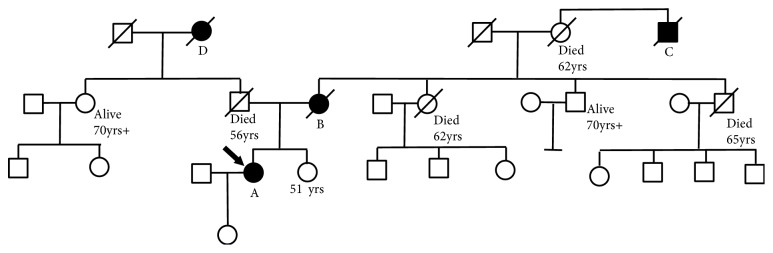
A: family pedigree. Proband for this case report is family member A. Other cancer-affected relatives: B-D, as described in the article.

The patient was reviewed in the Clinical Genetics service. The pedigree is shown in Figure 4. The patient (A on the pedigree) also had a past history of hysterectomy, with ovaries preserved, at the age of 31 for grade 3 cervical intraepithelial neoplasia, nonmelanomatous skin cancer, and lower limb squamous cell carcinoma in-situ (Bowen's disease). She reported that her late mother (B) had radiotherapy treatment for a cancer affecting a finger at the age of 10 years in the early 1950s, subsequently developed breast cancer at the age of 58, and died at the age of 67. The patient's maternal grandmother died at the age of 62 without cancer, but the brother (C) of that lady was reported to have died from a cancer affecting his leg, considered possibly a sarcoma. The patient's paternal grandmother (D) died of a cancer of unknown primary site aged 42.* TP53* gene screening was performed for the patient, identifying a heterozygous pathogenic mutation c.524G>A; p.(Arg175His), consistent with a diagnosis of Li Fraumeni syndrome. From the family history, inheritance was considered more likely to be from the proband's mother than father, although unconfirmed at present.

The patient was treated with three cycles of cyclophosphamide, doxorubicin, vincristine, and prednisolone (CHOP), followed by a further three cycles with the doxorubicin replaced with gemcitabine in view of previous anthracycline-exposure during her adjuvant breast cancer treatment. A post-treatment PET CT scan showed no remaining abnormal lesion or metabolic activity. Radiotherapy was not given.

## 3. Discussion

To our knowledge, this is the fifth case of BIA-ALCL in association with Li Fraumeni syndrome reported in the literature to date. This strengthens the evidence that this is a true, rather than chance, association. There have been two single case reports [9, 10]. In another series of BIA-ALCLs, germline* TP53* mutations were detected in 2 of 11 cases; both patients had previous personal and family history of breast cancer; in one of the two cases, a somatic “second hit” mutation was detected in the tumour, which is the same mutation that we detected in germline in our patient [11].

The mechanism of development of BIA-ALCL is unclear. It has been postulated that chronic inflammation due to a bacterial biofilm, more prevalent on textured implants, leads to T-cell hyperplasia and subsequent malignant transformation [12]. Somatic mutations leading to increased activation* JAK/STAT* signalling, particularly* STAT3* gene mutations, are common, suggesting that this is the main driver pathway in BIA-ALCL [11].

*TP53* mutations have been identified in some BIA-ALCL tumours [11, 13]. However,* TP53* mutations are not identified in the majority of BIA-ALCL, with either single or biallelic “hits”. Therefore, Li Fraumeni syndrome/germline* TP53* mutation may be a risk factor, rather than a driver for BIA-ALCL development. BIA-ALCL-derived cell line studies have shown evidence of dysregulation of p53 signalling pathways in response to DNA damage [14]. A high incidence of thymic lymphoma development was noted in a study of biallelic* TP53*-knockout mice [15]. There is also evidence that* TP53* can show haploinsufficiency in the heterozygous state [16]. Therefore, it is possible that BIA-ALCL develops due to inadequate tumour-suppressor activity in Li Fraumeni patients, allowing greater likelihood of clonal survival of lymphoma cells that have developed due to the implant-associated biofilm and cytokine environment.

In around 80% of cases, BIA-ALCL presents as effusion-limited disease, with nodal involvement being less common. In our patient, the presence of lymph node involvement at presentation may suggest more aggressive behaviour, possibly in the context of Li Fraumeni syndrome. However, pathological lymph node involvement was not reported in two other single detailed case reports [9, 10], so it is not possible to draw firm conclusions.

CHOP is a standard first-line chemotherapy for the management of T-cell lymphoma, but with ongoing investigation of use of etoposide (in CHOEP) and gemcitabine amongst other agents [17]. Our patient had some adjustment of standard treatment (second half of treatment using gemcitabine rather than doxorubicin) in view of previous anthracycline-exposure, still achieving complete response on functional imaging.

The interval between initial breast implant surgery and diagnosis with the BIA-ALCL was 14 years in this case, which is longer than described in other single detailed case reports in which there were intervals of 3 years [9] and 7 years [10]. It could be hypothesized that LFS-associated lymphomas would develop after a shorter median interval than non-LFS-associated. However, the interval range in general series is wide from 2 years to 32 years [1]. The number of reported cases in the literature confirmed as being associated with LFS is currently too small to draw firm conclusions.

The absolute risk of BIA-ALCL in Li Fraumeni patients is unknown. However, avoidance of textured implants in known or suspected* TP53* mutation carriers is prudent. Our patient is planned to have removal of the contralateral implant (on the previously radiotherapy-treated side).

It is important to assess personal and family history to identify potential Li Fraumeni cases amongst those affected with BIA-ALCL.
